# Heterozygous junctophilin-2 (*JPH2*) p.(Thr161Lys) is a monogenic cause for HCM with heart failure

**DOI:** 10.1371/journal.pone.0203422

**Published:** 2018-09-20

**Authors:** Sari U. M. Vanninen, Krista Leivo, Eija H. Seppälä, Katriina Aalto-Setälä, Olli Pitkänen, Piia Suursalmi, Antti-Pekka Annala, Ismo Anttila, Tero-Pekka Alastalo, Samuel Myllykangas, Tiina M. Heliö, Juha W. Koskenvuo

**Affiliations:** 1 Heart Center, Tampere University Hospital, Tampere, Finland; 2 Heart and Lung Center, Helsinki University Hospital, University of Helsinki, Helsinki, Finland; 3 Blueprint Genetics, Helsinki, Finland; 4 Faculty of Medicine and Life Sciences, University of Tampere, Tampere, Finland; 5 Divisions of Pediatric Cardiology, Children's Hospital/Helsinki University Hospital, Helsinki, Finland; 6 Department of Pediatrics, Tampere University Hospital, Tampere, Finland; 7 Department of Internal Medicine, Seinäjoki Central Hospital, Seinäjoki, Finland; 8 Institute of Biomedicine, University of Helsinki, Helsinki, Finland; 9 Department of Clinical Physiology and Nuclear Medicine, HUS Medical Imaging Center, Helsinki University Hospital and University of Helsinki, Helsinki, Finland; Scuola Superiore Sant'Anna, ITALY

## Abstract

During the last two decades, mutations in sarcomere genes have found to comprise the most common cause for hypertrophic cardiomyopathy (HCM), but still significant number of patients with dominant HCM in the family are left without molecular genetic diagnosis. Next generation sequencing (NGS) does not only enable evaluation of established HCM genes but also candidate genes for cardiomyopathy are frequently tested which may lead to a situation where conclusive interpretation of the variant requires extensive family studies. We aimed to characterize the phenotype related to a variant in the junctophilin-2 *(JPH2)* gene, which is less known non-sarcomeric candidate gene. In addition, we did extensive review of the literature and databases about *JPH2* variation in association with cardiac disease. We characterize nine Finnish index patients with HCM and heterozygous for *JPH2* c.482C>A, p.(Thr161Lys) variant were included and segregation studies were performed. We identified 20 individuals affected with HCM with or without systolic heart failure and conduction abnormalities in the nine Finnish families with *JPH2* p.(Thr161Lys) variant. We found 26 heterozygotes with the variant and penetrance was 71% by age 60 and 100% by age 80. Co-segregation of the variant with HCM phenotype was observed in six families. Main clinical features were left ventricular hypertrophy, arrhythmia vulnerability and conduction abnormalities including third degree AV-block. In some patients end-stage severe left ventricular heart failure with normal or mildly enlarged diastolic dimensions was detected. In conclusion, we propose that the heterozygous *JPH2* p.(Thr161Lys) variant is a new Finnish mutation causing atypical HCM.

## Introduction

Hypertrophic cardiomyopathy (HCM) is considered the most common form of inherited cardiomyopathies estimated to affect one in 500 in general population [1]. Diagnosis of HCM is made by two-dimensional echocardiography showing hypertrophied, non-dilated left ventricle (LV) in the absence of other cardiac or systemic causes of hypertrophy such as aortic valve stenosis or hypertension [2]. HCM may manifest at any age but typically in the adulthood. The clinical course of the disease varies significantly from person to person. Some patients remain asymptomatic through their life whereas others suffer from arrhythmias or embolic stroke, develop severe heart failure or experience sudden cardiac death (SCD) even at early age [3–5].

Hereditary HCM is a dominant disorder, commonly associated with mutations in sarcomere genes. Phenocopies of HCM include Anderson-Fabry disease (galactosidase alpha, *GLA*), Danon disease (lysosomal-associated membrane protein 2, *LAMP2)*, *PRKAG2* related glycogen storage disease (protein kinase AMP-activated non-catalytic subunit gamma 2, *PRKAG*2), cardiac amyloidosis, neuromuscular diseases and malformation syndromes (Noonan spectrum syndromes).[6] Genetic diagnostics has proven as an effective strategy to differentiate between potential underlying causes and to rule out phenocopies. Accurate molecular genetic diagnosis helps to detect the genetic cause of those phenocopies that might require special therapy (e.g. enzyme replacement therapy) [7–9].

The most established genes associating with HCM are myosin binding protein C (*MYBPC3)*, myosin heavy chain 7 *(MYH7)*, troponin I3, cardiac type *(TNNI3)*, troponin T2, *cardiac type (TNNT2)*, tropomyosin 1 *(TPM1)*, myosin light chain 2 *(MYL2)*, myosin light chain 3 *(MYL3) and* actin, alpha, cardiac muscle 1 *(ACTC1) [10].* The role of candidate genes such as troponin C1, slow skeletal and cardiac type (*TNNC1)* [11] and actinin alpha 2 *(ACTN2)* [12] has remained obscure. Diagnostic yield of molecular genetic testing in daily practice is 25–40% in HCM [10]. This demonstrates the challenge of differentiating genetic disease from acquired conditions or other systemic diseases and indicates that there are novel disease genes and more complex genetic variations left to be discovered. Although it has been difficult to demonstrate phenotypic correlations between different genes and different mutations, there is a significantly worse outcome in patients tested positive vs. negative for mutations in sarcomere genes [13]. A large proportion of the mutations are unique for a family and have not been reported before. In certain populations, however, founder mutations comprise a large part of detected mutations. To date HCM founder mutations have been identified in *MYBPC3*, *MYH7* and *TPM1* genes, and reported in the Netherlands [14], Spain [15], South Africa [16], Finland [17], Italy [18], Japan [19], South Asia [20] and in the Amish population [21].

Junctophilin-2 gene (*JPH2*) is the major structural protein in cardiomyocytes for coupling of transverse (T) tubule-associated L-type Ca^2+^ channels and type-2 ryanodine receptors on the sarcoplasmic reticulum within junctional membrane complexes (JMC) [22, 23]. Signaling between these two Ca^2+^ channels is required for normal cardiac contractility. Disruption of the JMC is a common finding in failing hearts. Downregulation of *JPH2* gene has been associated with heart failure and mutations in this gene have been suggested to associate with HCM. *JPH2* was initially published as candidate gene for HCM in 2007 when Matsushita *et al*. found p.(Gly505Ser) in four probands among 148 Japanese HCM patients [24] but this variant was later found to be a common polymorphism present in up 4–6% in Asian/African populations. Role of the JPH2 in cardiomyopathies has been obscure as only one rare variant segregating with any type of cardiomyopathy has been published [25].

In this study, we characterize the cardiac phenotype related to *JPH2* c.482C>A, p.(Thr161Lys) variant in nine Finnish index patients and their family members. We also review evidence gathered from the literature and variant databases that supports or is against the pathogenicity of the mutations in *JPH2* to highlight challenges we have when rare gene variants from candidate genes are detected.

## Subjects and methods

The methods for this trial is available as supporting information; see S1 Protocol http://dx.doi.org/10.17504/protocols.io.[PROTOCOL]

Index patients with the *JPH2* variant c.482C>A, p.(Thr161Lys) and their relatives from four Finnish hospitals were included. A written informed consent was obtained from all participants. This study has been approved by the Ethical Review Committee of the Department of Medicine, University of Helsinki (Dnro 307/13/03/01/11) and by the Ethical Review Committee of the Department of Medicine, University of Tampere (R08070) and conforms to the ethical principles outlined in the Declaration of Helsinki.

HCM was clinically diagnosed according to ESC Guidelines [2]. Hypertrophic cardiomyopathy (HCM) is defined by the presence of increased LV wall thickness that is not solely explained by abnormal loading conditions. In adults, the diagnosis of HCM requires LV wall thickness ≥15 mm as measured by any imaging technique. Correspondingly, the clinical diagnosis of HCM in first-degree relatives of patients with LVH ≥15 mm is based on the presence of otherwise unexplained increased LV wall thickness ≥13 mm. In children, the diagnosis of HCM requires LV wall thickness more than two standard deviations greater than the predicted mean.

Family history was obtained and pedigrees were drawn. The adult participants were assessed clinically at Heart and Lung Center, Helsinki University Hospital, at the Heart Hospital, Tampere University Hospital or at the Seinäjoki Central Hospital by physical examination, resting 12-lead ECG, appropriate laboratory tests and transthoracic echocardiography (TTE). Cardiac MRI was performed in some cases especially in patients with borderline diagnostic findings at echocardiography. All participants are of Finnish ethnicity.

### Molecular genetic studies

Genetic testing was carried out from genomic DNA using the OS-Seq™ (oligonucleotide-selective sequencing) next-generation sequencing method [26, 27]. The genetic evaluation of the index patients was performed using the Blueprint Genetics Core Cardiomyopathy or Pan Cardiomyopathy Panels covering 69 and 103 genes, respectively, associated with cardiomyopathies and their genetic phenocopies (Supplementary file 1). The presence of the variant in probands’ relatives was studied by bi-directional Sanger sequencing.

## Results

### Genetic studies

**Fig 1:**
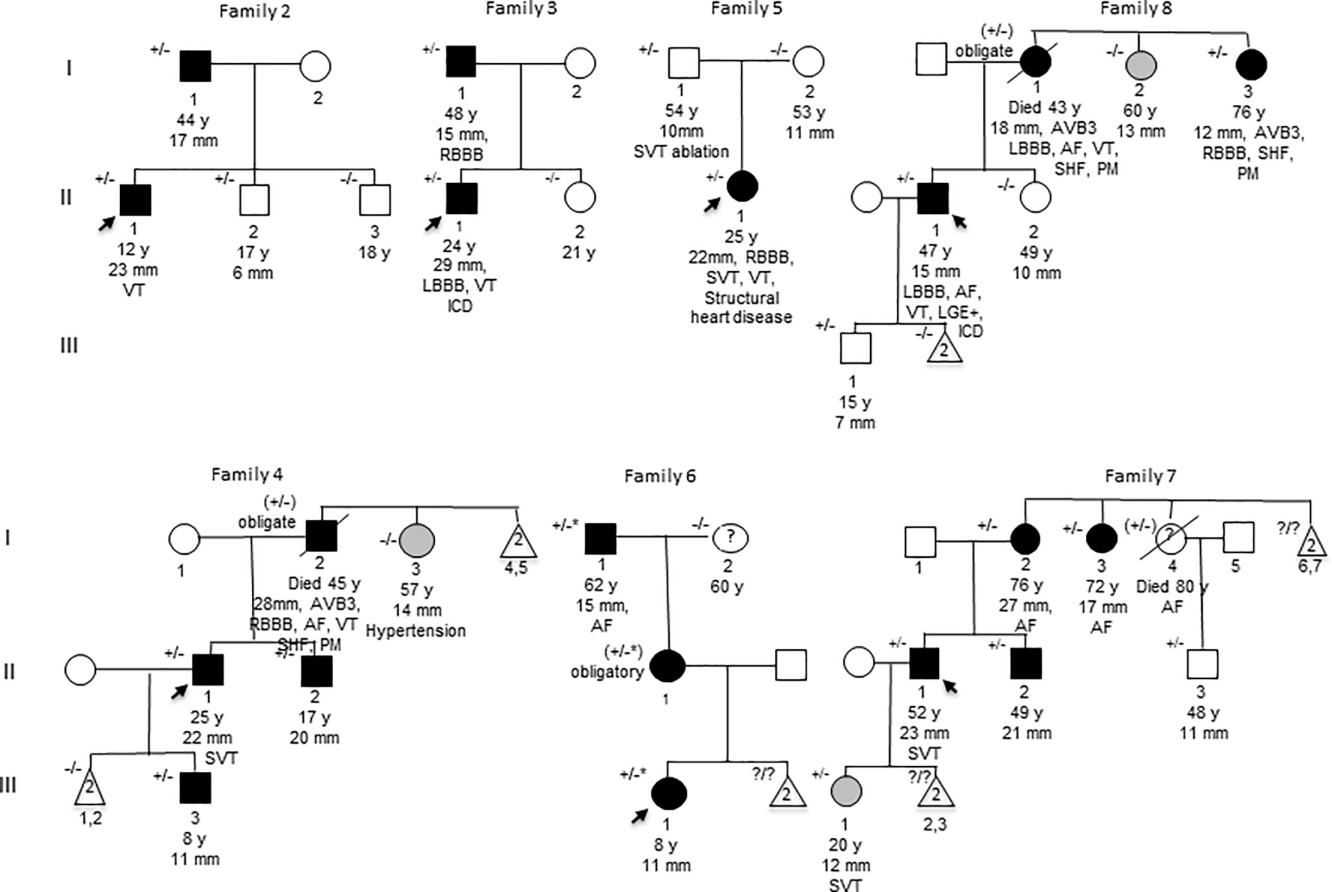
Pedigrees of seven families affected with the *JPH2* c.482C>A, p.(Thr161Lys), (rs587782951, NM_020433.4) variant. Pedigrees of three families affected with the *JPH2* c.482C>A, p.(Thr161Lys), (rs587782951, NM_020433.4) variant. Circles represent women, squares men and triangles gender blinded. Black-filled symbols represent individuals who fulfill ESC 2008 diagnostic criteria for HCM with [2]. We also considered as affected with HCM one family member (Family 8:I.3) who had imminent cardiomyopathy with borderline LVH (12 mm), 3-degree AV block, RBBB and severe systolic heart failure. Genotypes: +/- heterozygous for the *JPH2* p.(Thr161Lys), -/- wild type allele, * *MYBPC3* p.(Gln1061*). Age of the family members at last follow-up, maximum LV wall thickness and some other key signs of clinical disease are listed below the symbols. Arrows indicate index patients. Abbreviations listed in Table 1.

A heterozygous *JPH2* c.482C>A, p.(Thr161Lys) (NM_020433.4) variant was observed in nine unrelated Finnish probands with cardiomyopathy. Altogether the p.(Thr161Lys) was detected in 20 affected individuals. The variant co-segregated with HCM in six families (Families 2–4, 6–8, Fig 1) and in three families the mutation was found only in the probands (Family 9), in probands and young family member without HCM (Family 1) or in probands and in another family member without HCM (Family 5). Systolic heart failure or conduction abnormalities were observed in every family. These features were present in 12/20 (60%) of the affected patients including ten heterozygous affected individuals and two obligate carriers. The p.(Thr161Lys) was absent in three family members without LV hypertrophy who were over 20 years of age (3.I.2, 5.I.2, 8.II.2). It was also absent in asymptomatic 60-year old female (Family 6.I.2) who was not evaluated clinically and two individuals with LVH likely explained by severe hypertension (Family 4:I.3 and Family 8:I.2). Pedigrees of the families 2–8 are presented in Fig 1. This heterozygous missense variant has not been observed in the Exome Aggregation Consortium (ExAC) data set, comprised in total of over 60,000 unrelated individuals or in the Genome Aggregation Database (gnomAD; total of 126,216 exomes and 15,137 genomes). *In silico* bioinformatic tools Polyphen, SIFT and Mutation Taster predict it to be deleterious. Threonine is conserved amino acid at this position among mammals.

The main clinical characteristics of the probands and family members are shown in Table 1. These include left ventricular hypertrophy (LVH), and in some cases end-stage severe left ventricular (LV) failure with normal or mildly dilated LV. Average at diagnosis was 26.9±20.6 years in the nine probands and their maximum LV wall thickness was 20.4±5.2 mm at age of 34.1. Atrial or ventricular arrhythmias considered abnormal were observed in 13/20 (65%) of the heterozygotes. Pacemaker (PM) or ICD was implanted in 6/20 (30%) and reduced LVEF (<47%) or elevated proBNP concentration (>300 ng/l) was observed in 9/20 (45%) of the patients. Penetrance of HCM was 48%, 71% and 100% by age of 40, 60 and 80, respectively.

**Table 1:** Clinical characteristics of the probands and their family members.

Family	Age (M/F)	Genotype	Conduction defect	Arrhythmias	PM, ICD	LV- WT	LVEDD (mm)/ EF (%)	proBNP (ng/l)	Age at dg	Phenotype	Other
**Family 1**											
**I.1**	52M	+/-	no	AF	ICD	20	46/56%	400	47	HCM	
**Family 2**											
I.1	44M	+/-	no	no	no	16	46/72%	76	44	HCM	
**II.1**	12M	+/-	no	VT	no	23	48/77%	315	12	HCM	
II.2	16M	+/-	pRBBB	no	no	6	51/63%	NA	-	normal	
**Family 3**											
I.1	48M	+/-	RBBB	no	no	15	49/72%	44	41	HCM	
**II.1**	24M	+/-	LBBB	VT	ICD	29	44/75%	145	13	HOCM	Pk-grad 81mmHg
**Family 4**											
I.2	45M	n.a.	AVB3, LAHB, RBBB	AF, VT	PM	28	55/20%	8425	17	HOCM, SHF	Pk-grad 50mmHg Died 45 y
I.3	57F	-/-	no	no	no	14	43/60%	33	-	LVH/Hypertens	BP 160/110 mmHg
**II.1**	25M	+/-	no	SVT	no	22	45/67%	104	9	HCM	
II.2	17M	+/-	LAHB	no	no	20	49/55%	NA	1	HCM	
III.3	8M	+/-	no	no	no	11	35/>50%	NA	1	HCM	
**Family 5**											
I.1	54M	+/-	no	SVT	no	10	50/70%	NA	-	normal	SVT ablation
I.2	53F	-/-	no	no	no	11	44/83%	164	-	normal	
**II.1**	25F	+/-	RBBB, LAHB	VES, VT, SVT	no	22	43/60%	5700	12	HCM, SHF	Multiple VSDs, PDA operated aged 2 years. During pregnancy LVEF 45%
**Family 6**											
I.1	62M	+/-,*	LAHB	AF	no	15	40/52%	998	61	HCM	
I.2	60F	-/-	NA	NA	NA	NA	NA	NA	-	NA	
II.1	F	(+/-,*)	NA	NA	NA	NA	NA	NA	-	HCM	
**III.1**	8F	+/-,*	NA	no	no	11	33/>60%	3928	7	HCM	
**Family 7**											
I.2	76F	+/-	LAHB	AF	no	27	49/56%	NA	?	HCM	
I.3	72F	+/-	no	AF	no	17	45/60%	NA	?	HCM	
I.4	80F	(+/-)	no	AF	no	NA	NA	NA	?	?	Died age 80 y
**II.1**	52M	+/-	AVB1	VES, SVT	no	23	49/63%	NA	43	HCM	
II.2	49M	+/-	AVB1	no	no	21	60/50%	NA	?	HCM	
II.3	48M	+/-	no	no	no	11	NA	NA	-	normal	
III.1	20F	+/-	no	SVT	no	12	44/66%	NA	19	normal	
**Family 8**											
I.1	43F	(+/-)	AVB3, LBBB	AF, VT	PM	18	70/30%	6637	25	HCM, SHF	Died aged 43. HF. WT at autopsy 15 mm.
I.2	60F	-/-	no	no	no	13	40/71%	160	-	LVH/Hypertens	
I.3	76F	+/-	AVB3, LAHB, RBBB	no	PM	12	46/40%	5267	67	HCM	Mixed cardiomyopathy
**II.1**	47M	+/-	AVB1, LBBB	AF, VT	ICD	15	51/37%	4426	36	HCM, SHF	
II.2	49F	-/-	no	no	no	10	49/66%	31	-	normal	
III.1	15M	+/-	no	no	no	7	46/66%	NA	-	normal	
**Family 9**											
**I.1**	63F	+/-	no	AF	PM	19	45/60%	4082	63	HCM	

Index patients are marked in bold. Symbols and abbreviations: Age (M/F)–age and gender (M, male; F, female); Genotype–+/- is heterozygous and (+/-) obligatory heterozygous for p.(Thr161Lys) in *JPH2* and -/- is wild type, * heterozygous for *MYBPC3* Gln1061*; R/LBBB–presence of right/left bundle branch block; LAHB–left anterior hemiblock; AVB1-3 –atrioventricular block types 1–3; Arrhythmias–AF for atrial fibrillation/flutter, SVT for supraventricular tachycardia (>10 episodes short episodes per day or SVT requiring cardioversion), VT for ventricular tachycardia ≥ 3 beats with frequency >100/min, VES for ventricular extrasystoles >1000 per day, SVES for supraventricular extrasystoles >5000 per day; PM, ICD, CRT-P/D–pacemaker, implantable cardioverter-defibrillator, cardiac resynchronization therapy device; LV-WT-maximal left ventricular wall thickness measured by echocardiography or cardiac MRI; LVEDD & EF–left ventricular end-diastolic diameter (mm) and ejection fraction (%); Age at dg-age at diagnosis of cardiomyopathy; Phenotype–phenotype at diagnosis, HCM–hypertrophic cardiomyopathy, LVH–left ventricular hypertrophy; SHF–systolic hear failure; Other–other significant clinical features

#### Family 1

Proband of this family was diagnosed HCM at age 47. His genotype positive (not drawn on pedigree) son was phenotype negative with maximal wall thickness of 10 mm at age 22, which is in line with age-dependent penetrance of cardiomyopathies.

#### Family 2

Proband (II.1) of family 2 developed HCM at age of 12 and has significant burden of arrhythmias. Subsequently, his genotype positive father was found to have also HCM and arrhythmias. Proband’s brother was genotype positive yet phenotype negative at age of 16.

#### Family 3

Proband (II.1) developed HCM at age of 13 and LV wall thickness increased up to 29 mm by age 24. He had also LVOT obstruction with 81mmHg systolic gradient. The patient was prescribed beta blockers and disopyramide, and LVOT gradient decreased to 20mmHg. His symptoms, chest pain and shortness of breath presenting during light walking, were suppressed by the medication. His genotype positive father has also HCM but no arrhythmias. The proband has ventricular arrhythmias. Both affected individuals in this family have preserved systolic function.

#### Family 4

Proband (II.1) is a male with clinical diagnosis of HCM at age 9. He has had recurrent episodes of sustained atrial tachyarrhythmias. On echocardiography, maximal LV wall thickness was 22mm. Proband’s 17-year-old genotype positive brother (II.2.) had maximal LV thickness of 20mm. He and proband’s son were diagnosed at very early age. Proband’s father (I.2) was diagnosed with HCM aged 17 years as his ECG showed inferolateral T-inversions like the ECGs of his both sons and echocardiography revealed septal thickness of 17mm. Later he developed variable 2–3 degree AV block and received pacemaker. He had also LVOT obstruction with 50mmHg systolic gradient. The patient was prescribed beta blockers and disopyramide and LVOT gradient decreased to 21 mmHg. At age 26, his LVEF was 70%. During the follow-up, maximal septal thickness increased to 28mm and posterior wall to 20mm. The patient had paroxysmal atrial flutter and later chronic atrial fibrillation (AF). By the age of 40 years, the patient developed NYHA 3 dyspnea and his LVEDD was 58mm, LVEF 40% and septal thickness 18mm. He had right heart failure and ascites, proBNP was 8425ng/l. Aged 45 years LVEDD was 55mm and LVEF 20–25%. The patient was listed for heart transplantation but died aged 45 years due to heart failure and sepsis. He deceased before the NGS technology became to clinical use and his genotype remains unknown. His disease fits well with the phenotype described here in genotype positive individuals. Genotype of proband’s mother is also unknown, thus we were not able to confirm that the *JPH2* variant was inherited from the affected parent. Index patient’s father’s mother had also HCM and died aged 42 years while sleeping and her father (proband’s father’s maternal grandfather) died aged 45 years due to an assumed cardiogenic reason (not drawn in pedigree). As a whole probands and two adult family members heterozygous for the variant fulfilled the criteria for HCM, whereas one individual (I.3), who is negative for the variant has LVH likely explained by severe hypertension (blood pressure 160/110mmHg) and obesity (BMI 32 kg/m^2^).

#### Family 5

Proband (II.1) had multiple VSDs, mild mitral stenosis and patent ductus arteriosus necessitating surgery aged 2 years. HCM was diagnosed when she was 12. ECG showed RBBB likely due to previous cardiac surgery, abnormal amount of ventricular and atrial premature complexes and she also suffers from SVTs and VTs. She had high levels of pro-BNP (5700 ng/L) although having normal systolic function at echocardiography. Her genotype positive father has no morphological HCM phenotype but he underwent ablation for SVT with good response.

#### Family 6

Proband (III.1) is a girl who developed HCM by the age of 7 years. She has also a well-established pathogenic nonsense mutation in *MYBPC3* (c.3181C>T, p.(Gln1061*)). Her pro-BNP levels are high (3928 ng/L). She has inherited both of the variants from his mildly affected grandfather through her mother who has HCM and is obligate carrier of both variants.

#### Family 7

In this family, the index patient (II.1), his brother (II.2), mother (I.2) and mother’s sister (I.3) were heterozygous for the *JPH2* p.(Thr161Lys) variant. They all fulfilled the imaging criteria of HCM with maximal LV wall thicknesses of 17-27mm. None of these patients had heart failure. Mother’s other sister (I.4) who was an obligatory carrier died at age 80 due to mesenterial thrombosis. Her son is heterozygous for the *JPH2* variant. Twenty-year-old daughter (III.1) of the index has borderline phenotype (septal thickness 12 mm) and her ECG showed abnormal Q-waves at I, II, aVF, V5-6 leads and episodes of ectopic atrial rhythm.

#### Family 8

The index patient (II.1) is heterozygous for the *JPH2* p.(Thr161Lys) variant. He was a previously sportive male who presented with decreased exercise tolerance at the age of 36 years. ECG showed LVH and on echocardiography LV was 51/36mm, EF 55% and septum 14mm at maximum. Five years later septum measured 15mm fulfilling the HCM criteria. At age of 45, ECG showed sinus rhythm and trifascicular block (LBBB + type 1 AV block). TnT concentration was constantly increased up to 48 ng/l (normal range < 15ng/l) and proBNP concentration has increased up to 4426 ng/l. He has had paroxysmal atrial fibrillation (AF)/flutter and monomorphic ventricular tachycardia (VT). Coronary angiography was normal. Cardiac MRI demonstrated extensive late gadolinium enhancement (LGE) especially at septal and anterior regions. FDG-PET did not detect signs of inflammation. Endomyocardial biopsies from left and right ventricles remained non-diagnostic and showed mainly fibrosis. Electrophysiologic study demonstrated VT. On echocardiography, LV was 51mm, septum 14mm, PW 12mm and EF 37%. Hemodynamics were restrictive. The patient has received an ICD. His mother (I.1) was an obligatory carrier of the variant. She presented with loss of weight, fainting and palpitations aged 28 years and ECG showed LBBB. In cardiac catheterization, LVEDP was elevated and left ventricular cineangiography (LV-cine) showed stiff septum, compatible with HCM. Eight years later she was examined due to tachycardias and collapse episodes. Maximum LV wall thickness was 18 mm. In LV-cine, EF was 32%, stroke volume 38ml. The patient received pacemaker due to episodes of syncope and tachyarrhythmia. At age 40 she had atrial fibrillation and 3-degree AV block and at age 42 her LVEDD was 70 mm and EF 30%. She was treated with digoxin, furosemide, amiodarone, atenolol, captopril and warfarin. Cardiac CT showed dilated left atrium and ventricle but normal pericardium. Before death at the age of 43, she had heart failure (HF) and repeated VT’s. Autopsy showed fibrotic changes especially at endocardium of posterior and septal walls. There was very thin and fibrotic area with a size of 3x4 cm in the anterior LV wall. Maximum wall thickness in autopsy was 15mm. Clinical diagnoses comprised cardiomyopathy, endocardial and myocardial fibrosis. No material for genotype analysis was available. Mother’s sister (I.3) is also heterozygous for the p.(Thr161Lys) variant and was considered affected as she has imminent cardiomyopathy with borderline LVH (12 mm), AF, 3-degree AV block, RBBB and severe systolic heart failure not explained by other causes. She has received a PM. Proband’s non-carrier aunt (I.2) had mild LVH likely due to hypertension. Proband’s non-carrier brother (II.2) had normal findings in cardiac evaluations as well as proband’s genotype positive son (III-1) who was 15 years old at last follow-up.

## Discussion

We have identified the *JPH2* p.(Thr161Lys) variant in nine Finnish index patients with HCM and have shown co-segregation of the variant with cardiomyopathy in six of these families. In the other three, only the index person presented HCM phenotype due to the size of the family or the young age of the variant carriers. This is the first *JPH2* variant shown to be causative for HCM.

For almost twenty years data has accumulated on the role *JPH2* in cardiac physiology. However, little is known about the significance *JPH2* as a causative gene for cardiomyopathy. *JPH2* is a cardiac specific member of the junctophilins and it has emerged as a potentially important regulator of excitation-contraction coupling in cardiomyocytes. Several studies have highlighted the importance of *JPH2* for normal cardiac physiology [23, 28]. Mice with acute conditional cardiac specific knockdown of *JPH2* have a high incidence of mortality with a rapid development of systolic heart failure [29]. These mice demonstrated grossly enlarged hearts with dilated ventricles and reduced systolic function on echocardiogram. In another mouse model study, heterozygous *JPH2* p.(Glu169Lys) mice had a higher incidence of pacing-induced AF secondary to abnormal spontaneous Ca^[2+]^ waves and increased spark frequency [30].

Cardiac phenotype related to this variant differs somewhat from typical HCM. Two Finnish founder mutations for HCM have previously been published one in myosin binding protein C (*MBPC3*) gene and the other in alpha tropomyocin (*TPM1*) gene [17, 31]. The *JPH2* p.(Thr161Lys) variant presented here differs from the other two in clinical presentations. The *JPH2* variant associates with earlier disease onset (27 years vs. 52 and 49 years) than the *MYBPC3* p.(Gln1061*) or *TPM1* p.(Asp175Asn), respectively, although no patients under age of 16 were included in the previous study [17]. Systolic heart failure was observed in 5 (25%) and LV dysfunction was present in half of the affected patients with *JPH2* variant when defined by EF<47% or elevated proBNP. Of the probands with *MYBPC3* p.(Gln1061*), none has had congestive heart failure. However, dyspnea was present in 31%. Also none of the index patients with the *TPM1* p.(Asp175Asn) mutation had significant systolic dysfunction (EF<46%) and only one (5%) had a history of systolic heart failure. Severe conduction defects defined by 3-degree AV-block or R/LBBB was observed in nine (45%) and AF in nine (45%) of the affected individuals or obligate carriers with *JPH2 variant*, but no SCDs were detected in our families. Of the individuals with *MYBPC3* p.(Gln1061*) variant, 26% presented syncope/pre-syncope, 23% had either chronic or paroxysmal atrial fibrillation, 9% have had sustained VT or ventricular fibrillation and 26% had family history of SCD. Three out of 34 (9%) HCM patients carrying the *TPM1* p.(Asp175Asn) presented with a documented SCD at young or middle age [31]. Additionally, life-threatening arrhythmias were induced with programmed ventricular stimulation (PVS) in one third of patients carrying this mutation. Of the probands with *TPM1* or *MYBPC3* founder mutation, ICD was implanted in 2 (10%) and 7 (20%) patients respectively and no significant conduction problems necessitating pacemaker implantation where described in the index publications. These observations highlight the significant differences in clinical presentation of the patients with the previously characterized founder mutations compared to proposed *JPH2* mutation described in this study. However, the degree of LV hypertrophy may be similar in patients with these founder mutations (22±5 mm, 18±6 mm and 20±5 mm with variant in *MYPBC3*, *TPM1* or *JPH2*, respectively), although the JPH2 variants carriers were younger at a time of evaluation compared to carriers of other founder mutations. It should be still keep in mind that significant variation in clinical presentation within and between the families with the same mutation exists in all types of HCM.

There are altogether 19 *JPH2* variants associating with dilated or hypertrophic cardiomyopathy listed in the HGMD (Qiagen) and ClinVar databases (July 8, 2017) presented in Table 2. Seventeen of them are missense variants. Before this study, no convincing evidence of segregation within large pedigrees except for the p.(Glu85Lys) [25] and no *de novo JPH2* mutations have been reported in patients with HCM. In 2007, Landstrom *et al*. found two rare missense and one frameshift variant in *JPH2* in three probands with HCM but their families were not studied or genotyped [28]. In 2016, Sabater-Molina showed segregation of the *JPH2* p.(Glu85Lys) with dilated cardiomyopathy with or without left ventricular non-compaction cardiomyopathy (LVNC) features in a large family [25]. Furthermore, Quick *et al* have recently published *JPH2* p.(Ala405Ser) variant in a single patient with basal septal hypertrophy and diastolic dysfunction [32]. Most of the previously published variants are absent or rare in ExAC or gnomAD reference populations and thus they have potential to be disease causing. Truncating *JPH2* variants are relatively rare in ExAC reference population (carrier frequency 1 per 6,030 individuals) but due to small size of the gene, the pLI value is 0.01 which does not suggest that loss of function alterations would be poorly tolerated. The clinical data on HCM related to the previously published *JPH2* missense variants is limited, mostly clinical HCM appears to be diagnosed after teenage but at least the patient with p.(Glu169Lys) exhibited HCM already at the age of 5 months [30], similarly as two patients in this study.

**Table 2:** All *JPH2* variants described in literature and rare *JPH2* variants submitted to ClinVar Database with phenotype information.

*JPH2* Variant (NM_020433.4)	Pheno	gnomAD	PP	MT	Index (n)	Family (n)	Co-seg	Class	Other	Reference
c.253G>A, Glu85Lys	DCM	0/0	D	D	1	9	Yes	LP	Penetrance 78%	(Sabater-Molina et al. 2016)
c.301A>C, Ser101Arg	HCM	0/0	PD	B	1	0	No	VUS		(Landstrom et al. 2007)
c.421T>C, Tyr141His	HCM	0/0	D	D	1	0	No	VUS		(Landstrom et al. 2007)
c.494C>T, Ser165Phe	HCM	0/0	D	D	1	0	No	VUS		(Landstrom et al. 2007)
c.502A>C, Ser168Arg	HCM	0/0	D	D	1	0	Np	VUS		ClinVar 222654
c.505G>A, Glu169Lys	HCM	0/0	D	D	1	1	?	VUS		(Beavers et al. 2013)
c.559G>A, Gly187Ser	HCM	1/0	PD	B	1	0	No	VUS		ClinVar 222655
c.565G>A, Ala189Thr	SUD	94/0	B	B	1	0	No	LB		(Narula et al. 2015)
c.692G>A, Arg231Gln	Sdr	39/0	D	B	1	0	No	LB		(Farwell et al. 2015)
c.723C>G, Ser241Arg	DCM	0/0	D	D	1	0	No	VUS		ClinVar 155801
c.1013A>G, Glu338Gly	SCD	0/0	D	D	1	0	No	VUS	Enlarged RV	(Neubauer et al. 2016)
c.1213G>T, Ala405Ser	HCM	3/0	D	D	1	0	No	VUS	Ala405Thr 18 het gnomAD, *De novo*?	(Beavers et al. 2013)
c.1227C>G, Asn409Lys	DCM	0/0	PD	D	1	0	No	VUS		ClinVar 222656
c.1282C>T, Gln428*	DCM	13/0	-	-	1	0	No	VUS		ClinVar 222657
c.1513G>A, Gly505Ser	HCM	589/7	B	B	4	0	No	Benign		(Matsushita et al. 2007, Manrai et al. 2016)
c.1540G>A, Gly514Ser	LVNC	1/0*	D	D	1	0	No	VUS		ClinVar 222658
c.1564C>T, Arg522Trp	DCM	0/0	D	D	1	0	No	VUS		ClinVar 222659
c.1750C>A, Gln584Lys	LVNC	1/0*	B	B	1	0	No	VUS		ClinVar 180592
c.2011-1G>T	HCM	0/0	-	-	1	0	No	VUS		(Xu et al. 2015)

Abbreviations: Pheno, phenotype; gnomAD, number of heterozygotes/homozygotes in the gnomad reference population consisting 120,000 individuals; PP, PolyPhen; MT, MutationTaster; D, probably damaging in PolyPhen and disease causing in MutationTaster; PD, possibly damaging; B, benign in PolyPhen and polymorphism in MutationTaster; Index, number of index patients, Family, number of affected family members with the same variant as index patient; Co-seg, co-segregation; Class, classification of the variant by the authors relying on ACMG recommendation; LP, likely pathogenic; VUS, variant of uncertain significance; LB, likely benign; SUD, Sudden unexplained death; Sdr, complex syndrome

*, Non-pass (low quality) variant in gnomAD; LVNC, left ventricular non-compaction cardiomyopathy. Other abbreviations as in Table 1.

We identified one family (Family 6) where all three affected individuals carry the well-established pathogenic *MYBPC3* variant, p.(Gln1061*), in addition to *JPH2* p.(Thr161Lys) variant. Their phenotypic presentation did not differ significantly from the other families which are surprising as patients with homozygosity/compound heterozygosity or digenic pathogenic variants in sarcomere genes generally present at very early age [33–36]. In literature at least nine patients have been described who had homozygous or confirmed compound heterozygous disease causing variant in *MYBPC3* and at least the other variant is not truncating. In these patients the mean age at onset was 4.1 years. Three of these cases presented at neonatal phase, of which all died before age of two months [34, 35]. All patients with homozygous or compound heterozygous truncating pathogenic mutations in *MYBPC3* reported so far (n = 21) were diagnosed with severe cardiomyopathy and/or died within the first few months of life [35]. At the moment, it is unclear whether compound mutation present in sarcomere and non-sarcomere gene simultaneously have additive detrimental effect on disease onset and/or progression.

In conclusion, the *JPH2* p.(Thr161Lys) is classified as pathogenic based on ACMG classification scheme [37], as the variant resides in a conserved position, is predicted to be deleterious by *in silico* prediction tools, is absent in control populations and co-segregates with dominant HCM in six families. These observations strengthen the role of non-sarcomeric *JPH2* as a causative gene for HCM with or without systolic heart failure and conduction abnormalities. Further research is warranted to evaluate *JPH2* variants and other genes related to Ca^2+^ -handling in the cardiomyocyte to further shed light on the genetic background of HCM. Variant interpretation and correlation to phenotype is still challenging as earlier studies form major pitfalls by false classifications related to small reference populations, co-incidental segregations and evaluation of only a small subset of the potentially meaningful genes behind a patient’s phenotype. Large-scale genetic research will eventually bring more consistency to the evaluation of families with inherited cardiac diseases. Unfortunately, most of the submissions of the *JPH2* variants in ClinVar reviewed in this work did not include phenotype data and thus provide limited information for the clinical society. Variant sharing with relevant information of the phenotype in mutation databases is critically important to develop the field further on.

## Supporting information

S1 Protocol Genetic panels used for the family probands. Probands of each family were evaluated by either Blueprint Genetics Core Cardiomyopathy Panel (Family 1, 2, 3, 6, 7) or Pan Cardiomyopathy Panel (Family 4, 5, 8, 9). These next generation sequencing (NGS) panels are targeted into all protein coding exons and exon-intron boundaries of all target genes (listed below). These panels are validated to detect single nucleotide substitutions and small insertions, deletions and indels up to 46 bp. The panels have ISO 15189 and CAP accreditation.
(DOCX)
