# Supplementary material for: Heterozygous junctophilin-2 (JPH2) p.(Thr161Lys) is a monogenic cause for HCM with heart failure
Source: PLoS One. 2018 Sep 20;13(9):e0203422. doi: 10.1371/journal.pone.0203422 (PMC6147424; doi:10.1371/journal.pone.0203422)
Supplement: S1 Protocol — Probands of each family were evaluated by either Blueprint Genetics Core Cardiomyopathy Panel (Family 1, 2, 3, 6, 7) or Pan Cardiomyopathy Panel (Family 4, 5, 8, 9). These next generation sequencing (NGS) panels are targeted into all protein coding exons and exon-intron boundaries of all target genes (listed below). These panels are validated to detect single nucleotide substitutions and small insertions, deletions and indels up to 46 bp. The panels have ISO 15189 and CAP accreditation. (DOCX) [file pone.0203422.s001.docx]

**S1 Protocol. This is the S1 Protocol Title.** Genetic panels used for the family probands

Probands of each family were evaluated by either Blueprint Genetics Core Cardiomyopathy Panel (Family 1, 2, 3, 6, 7) or Pan Cardiomyopathy Panel (Family 4, 5, 8, 9). These next generation sequencing (NGS) panels are targeted into all protein coding exons and exon-intron boundaries of all target genes (listed below). These panels are validated to detect single nucleotide substitutions and small insertions, deletions and indels up to 46 bp. The panels have ISO 15189 and CAP accreditation.

**Core Cardiomyopathy Panel (72 genes)**: ABCC9, ACTC1, ACTN2, ANKRD1, BAG3, CALR3, CAV3, CRYAB, CSRP3, CTF1, CTNNA3, DES, DMD, DNAJC19, DNM1L, DSC2, DSG2, DSP, DTNA, EMD, EYA4, FHL1, FHL2, FKTN, FXN, GATAD1, GLA, HFE, ILK, JPH2, JUP, LAMA4, LAMP2, LDB3, LMNA, MIB1, MYBPC3, MYH6, MYH7, MYL2, MYL3, MYLK2, MYOM1, MYOZ2, MYPN, NEBL, NEXN, PDLIM3, PKP2, PLN, PRKAG2, PSEN1, PSEN2, RBM20, RYR2, SCN5A, SDHA, SGCD, TAZ, TCAP, TGFB3, TMEM43, TMPO, TNNC1, TNNI3, TNNT2, TPM1, TRIM63, TTN, TTR, TXNRD2, VCL.

**Pan Cardiomyopathy Panel (103 genes)**: ABCC9, ACADVL, ACTC1, ACTN2, AGL, ANKRD1, ATP5E, BAG3, BRAF, CALR3, CASQ2, CAV3, CBL, COA5, CRYAB, CSRP3, CTF1, CTNNA3, DES, DMD, DMPK, DNAJC19, DNM1L, DOLK, DSC2, DSG2, DSP, DTNA, EMD, EYA4, FHL1, FHL2, FKTN, FOXRED1, FXN, GAA, GATAD1, GLA, GLB1, GUSB, HFE, HRAS, ILK, JPH2, JUP, KRAS, LAMA4, LAMP2, LDB3, LMNA, MAP2K1, MAP2K2, MRPL3, MIB1, MYBPC3, MYH6, MYH7, MYL2, MYL3, MYLK2, MYOM1, MYOZ2, MYPN, NEBL, NEXN, NRAS, PDLIM3, PKP2, PLN, PRKAG2, PSEN1, PSEN2, PTPN11, RAF1, RBM20, RYR2, SCN5A, SCO2, SDHA, SGCD, SHOC2, SLC25A3, SOS1, SPRED1, SYNE1, SYNE2, TAZ, TCAP, TGFB3, TMEM43, TMEM70, TMPO, TNNC1, TNNI3, TNNT2, TPM1, TRIM63, TSFM, TTN, TTR, TXNRD2, VCL, XK
